# Immuno-PET Imaging to Assess Target Engagement: Experience from ^89^Zr-Anti-HER3 mAb (GSK2849330) in Patients with Solid Tumors

**DOI:** 10.2967/jnumed.118.214726

**Published:** 2019-07

**Authors:** C. Willemien Menke-van der Houven van Oordt, Adam McGeoch, Mats Bergstrom, Iain McSherry, Deborah A. Smith, Matthew Cleveland, Wasfi Al-Azzam, Liangfu Chen, Henk Verheul, Otto S. Hoekstra, Danielle J. Vugts, Immanuel Freedman, Marc Huisman, Chris Matheny, Guus van Dongen, Sean Zhang

**Affiliations:** 1 Department of Medical Oncology, Amsterdam UMC, Vrije Universiteit Amsterdam, Cancer Center Amsterdam, Amsterdam, The Netherlands; 2 Division of Experimental Medicine and Immunotherapeutics, Department of Medicine, University of Cambridge, Cambridge, United Kingdom; 3 OMID Molecular Imaging Consultancy, Uppsala, Sweden; 4 Clinical Pharmacology, Science, and Study Operations, GlaxoSmithKline, Uxbridge, United Kingdom; 5 Clin Pharm Advantage, LLC, Durham, North Carolina; 6 Bioimaging, Platform Technology and Science, GlaxoSmithKline, Stevenage, United Kingdom; 7 Biopharm Product Development and Supply, GlaxoSmithKline, King of Prussia, Pennsylvania; 8 Drug Metabolism and Pharmacokinetics, GlaxoSmithKline, King of Prussia, Pennsylvania; 9 Department of Radiology and Nuclear Medicine, Amsterdam UMC, Vrije Universiteit Amsterdam, Amsterdam, The Netherlands; 10 Freedman Patent, Harleysville, Pennsylvania; 11 Oncology R&D, GlaxoSmithKline, King of Prussia, Pennsylvania; and; 12 Hengrui Therapeutics, Inc., Princeton, New Jersey

**Keywords:** immuno-PET, HER3, antibody, target engagement, dose selection

## Abstract

PET imaging with radiolabeled drugs provides information on tumor uptake and dose-dependent target interaction to support selection of an optimal dose for future efficacy testing. In this immuno-PET study of the anti–human epidermal growth factor receptor (HER3) mAb GSK2849330, we investigated the biodistribution and tumor uptake of ^89^Zr-labeled GSK2849330 and evaluated target engagement as a function of antibody mass dose. **Methods:** ^89^Zr-GSK2849330 distribution was monitored in 6 patients with HER3-positive tumors not amenable to standard treatment. Patients received 2 administrations of ^89^Zr-GSK2849330. Imaging after tracer only was performed at baseline; dose-dependent inhibition of ^89^Zr-GSK2849330 uptake in tumor tissues was evaluated 2 wk later using increasing doses of unlabeled GSK2849330 in combination with the tracer. Up to 3 PET scans (2 hours post infusion [p.i.] and days 2 and 5 p.i.) were performed after tracer administration. Biodistribution and tumor targeting were assessed visually and quantitatively using SUV. The 50% and 90% inhibitory mass doses (ID_50_ and ID_90_) of target-mediated antibody uptake were calculated using a Patlak transformation. **Results:** At baseline, imaging with tracer showed good tumor uptake in all evaluable patients. Predosing with unlabeled mAb reduced the tumor uptake rate in a dose-dependent manner. Saturation of ^89^Zr-mAb uptake by tumors was seen at the highest dose (30 mg/kg). Despite the limited number of patients, an exploratory ID_50_ of 2 mg/kg and ID_90_ of 18 mg/kg have been determined. **Conclusion:** In this immuno-PET study, dose-dependent inhibition of tumor uptake of ^89^Zr-GSK2849330 by unlabeled mAb confirmed target engagement of mAb to the HER3 receptor. This study further validates the use of immuno-PET to directly visualize tissue drug disposition in patients with a noninvasive approach and to measure target engagement at the site of action, offering the potential for dose selection.

See an invited perspective on this article on page 899.

The human epidermal growth factor receptor (HER) family of receptor tyrosine kinases, also called ErbB, consists of 4 members: HER1 (epidermal growth factor receptor [EGFR], or ErbB1), HER2 (ErbB2), HER3 (ErbB3), and HER4 (ErbB4) (1). These receptors are important targets for rational drug design (1–3). Investigations have traditionally focused on the first 2 HER family members (EGFR and HER2), but growing evidence has also revealed a role for HER3 in driving malignant growth (4–6). HER3 overexpression has been shown to increase tumorigenesis in pancreatic adenocarcinoma (5) and is significantly associated with tumor progression and poor prognosis in patients with gastric cancer (6). A metaanalysis of HER3 expression and survival in solid tumors found that over half of patients with melanoma, cervical cancer, and ovarian cancer showed HER3 overexpression (7). Rates of overexpression in colorectal, gastric, and breast cancer ranged from 20% to 60% (7). HER3 somatic mutations have been identified in 11% of colon and gastric cancers, including several hot-spot mutations, which promote oncogenic signaling in the presence of HER2 (8). These findings suggest that HER3 can become an important therapeutic drug target (9,10).

GSK2849330 is a fully human monoclonal antibody (mAb) specific to HER3 that has enhanced antibody-dependent cell-mediated cytotoxicity and complement-dependent cytotoxicity mechanisms because of its high binding affinity to human Fc-γ-receptor RIIIa (responsible for initiating antibody-dependent cell-mediated cytotoxicity) and human complement protein C1q (responsible for initiating complement-dependent cytotoxicity) (10,11). Preclinical investigations with ^89^Zr-labeled GSK2849330 PET imaging in human tumor-bearing mice found dose-dependent, saturable uptake in HER3-positive CHL-1 tumors (12). A first-time-in-human phase 1 study (HER117158) recently investigated the pharmacokinetic properties, safety, and tolerability of GSK2849330 in participants with advanced HER3-positive tumors (NCT01966445; not published at time of writing). However, the optimal biologically active GSK2849330 dose has not been identified, as mAbs do not generally induce significant toxicity identifying a maximum tolerated dose.

Assuming that optimal therapeutic activity of mAbs requires tumor accumulation and saturation of target receptor binding, an understanding of the tumor-targeting capabilities of GSK2849330 and its interaction with the HER3 receptor would help identify an optimal dose sufficient to fully saturate tumor HER3 receptors and block HER3-mediated signaling. Immuno-PET with ^89^Zr-labeled antibodies allows for in vivo, whole-body analyses of mAb biodistribution, tumor targeting, and accumulation (13). ^89^Zr has a half-life of 3.3 d, allowing imaging over a period relevant to mAb biodistribution. By repeated PET scanning, visual and quantitative assessment of (heterogeneous) antibody binding to its target receptor can be determined over time throughout the body. This method has previously been used to investigate potential patient selection for mAbs in clinical use and to better understand novel mAbs used in phase 1 trials (14).

On target binding, internalization via endocytosis and subsequent lysosomal antibody degradation causes ^89^Zr to be retained within the cell. Thus, the recorded tissue signal on PET can be related to the cumulative exposure of bound antibody over time (area under the concentration–time curve [AUC]).

In practice, low doses of radiolabeled antibodies limit exposure and side effects (15) while allowing for optimal tumor interaction (16), therefore establishing a baseline for uptake. Coadministration of increasing doses of unlabeled mAb may prolong exposure but results in competition for receptor binding leading to decreased relative uptake of labeled mAb. The degree of target saturation can be established by comparing tracer uptake to initial baseline scans. The signal observed on PET imaging has 2 components: receptor-mediated, specific uptake and nonspecific tissue residence due to the presence of plasma and extracellular fluid in the tumor lesion. These components can be separated by Patlak transformation, which requires at least 2 imaging time points after tracer administration (15). Thus, the dose dependency of receptor-mediated clearance at a given site can be characterized by assessing the effect of different dose levels of unlabeled antibody. A prediction of the antibody dose required to saturate the target at the site of interest can then be derived.

The aim of the present study was to characterize the biodistribution and dose-receptor occupancy relationship of GSK2849330 labeled with ^89^Zr, in patients with advanced HER3-expressing solid tumors. We hypothesized that baseline and on-treatment PET imaging can predict the mass dose of therapeutic mAbs required for HER3 saturation on tumor lesions in patients.

## MATERIALS AND METHODS

This was a phase 1, open-label, single-center PET imaging study of the anti-HER3 mAb GSK2849330, labeled with ^89^Zr, in patients with advanced solid tumors expressing HER3 (ClinicalTrials.gov identifier NCT02345174; initiation, March 19, 2015; completion, June 2, 2016).

The study was approved by the appropriate regulatory and ethics committee (Amsterdam UMC, location VUMC, Medisch Ethische Commissie), was conducted in accordance with the International Conference on Harmonisation for Good Clinical Practice and the principles of the Declaration of Helsinki (17,18), and was monitored by an Internal Safety Review Committee. All participants provided written informed consent before study participation.

### Preparation of ^89^Zr-GSK2849330

^89^Zr-NCS-Bz-DFO-GSK2849330 (^89^Zr-GSK2849330) was produced as previously reported (12,19) and formulated to an infusion dose of 37 MBq (8 mg, 20 mL). The mean radiochemical purity assessed by spin filter was 99.8% ± 0.1%. The mean radiochemical purity was 100%, and the mean protein integrity was 99.4% ± 0.5%, as determined by size-exclusion high-performance liquid chromatography. The mean immune reactive fraction, assessed by a binding assay using HER3-coated plates, was 90.5% ± 4.1%. Sterility of each ^89^Zr-GSK2849330 batch was ensured by performing a medium fill immediately after final filter sterilization of each batch. These procedures resulted in a sterile final product with endotoxin levels of less than 0.3 EU/mL. For the experimental work requiring additional mAb, antibody without ^89^Zr or deferoxamine was used.

### Patients

Enrolled patients were aged at least 18 years and had a histologically confirmed diagnosis of HER3-positive solid tumors not amenable to standard treatment and an Eastern Cooperative Oncology Group performance status of 0 or 1, with good organ function. HER3 positivity was defined as documented HER3 expression on the surface of invasive tumor cells (archival tissue or fresh biopsy) determined by staining intensity (1+ [weak], 2+ [moderate], or 3+ [strong]) using an analytically validated immunohistochemistry assay (Ventana Medical Systems, Inc.). The H-score method was used to quantify HER3 positivity on a scale of 0–300 using the formula 1 × (percentage of weakly stained cells) + 2 × (percentage of moderately stained cells) + 3 × (percentage of strongly stained cells). Key exclusion criteria included leptomeningeal or brain metastases, prior HER3-directed treatment, significant cardiovascular risk, and evidence of another active malignancy. Anonymized individual participant data and study documents can be requested for further research from www.clinicalstudydatarequest.com.

### Study Design

**FIGURE 1.**
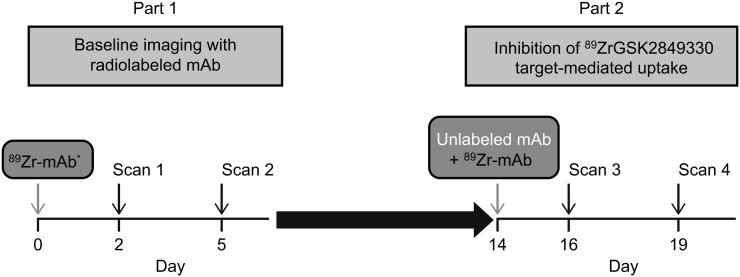
Study overview. *2-h scan for first 3 patients.

This 2-part imaging study was run in a staggered fashion with the first-time-in-human study of GSK2849330. In part 1, all immuno-PET imaging assessments were performed before treatment continuation with unlabeled GSK2849330 in part 2 (Fig. 1). Study enrollment was capped at 6 patients by the sponsor, after achievement of the primary objective (characterization of in vivo biodistribution). In part 1, patients received a tracer-only dose of 37 MBq of ^89^Zr-GSK2849330 (8-mg or 24-mg mass dose mAb) by intravenous infusion on day 0 (dose 1). On day 14, a variable total 24–30 mg/kg dose of unlabeled mAb was administered over 1 h by intravenous infusion, followed by a second dose of ^89^Zr-mAb (dose 2) within 1 h after the end of the infusion of unlabeled mAb.

The results of the first 2 patients were used to guide the baseline mass dose, with the consideration that if the mass dose was too low, nonspecific, or specific but non–tumor-related, binding would decrease systemic exposure, leading to less drug–target binding at the tumor site (sink effect).

Response was evaluated according to Response Evaluation Criteria in Solid Tumors, version 1.1, with the first analysis 12 wk after the start of part 2 and subsequently every 8 wk.

### Objectives/Endpoints

The primary objective was to measure the in vivo biodistribution of ^89^Zr-GSK2849330 in patients with HER3-positive tumors. The secondary objectives were to establish the dose dependency of inhibition of target-mediated uptake of ^89^Zr-GSK2849330 by unlabeled mAb, further characterize GSK2849330 pharmacokinetics, measure the dosimetry of ^89^Zr-GSK2849330, and assess safety, tolerability, and immunogenicity.

The primary endpoints were quantitative parameters derived from PET/CT images to assess uptake in tumor and normal tissues of interest, reported with SUV for each volume of interest (VOI; SUV_peak_ for tumor lesions and SUV_mean_ for normal tissues and organs). Secondary endpoints included pharmacometric modeling of tumor uptake, dosimetry, GSK2849330 pharmacokinetics, and safety.

### PET Imaging

Whole-body PET low-dose CT and ^89^Zr-PET scans were acquired on a European Association of Nuclear Medicine Research Ltd.–accredited Gemini TF-64 or Ingenuity TF PET/CT scanner (Philips Healthcare), as previously described (20). In the first 3 patients, PET/CT scans were acquired at 2 h (for dosimetry purposes), 48 h (day 2), and 120 h (day 5) after injection in the baseline study and at 48 h (day 16) and 120 h (day 19) after injection after the second tracer administration. In all remaining patients, scans were acquired 48 and 120 h after injection for each tracer administration (Fig. 1). PET scans, including whole-body coverage from brain to thighs, were acquired at 5 min per bed position.

### Image Analysis

Tumor accumulation of ^89^Zr-GSK2849330 was assessed by a physician experienced in PET image analysis and defined as visually enhanced accumulation exceeding local background. In all ^89^Zr PET scans, tumor VOIs were manually delineated and SUV_peak_ for lesions was derived using proprietary software (20). Only tumor lesions measuring at least 2 cm were quantified, to avoid partial-volume effects (20). The biodistribution of ^89^Zr-GSK2849330 to liver, spleen, kidney, lung, bone marrow, and blood was quantified using SUV_mean_. VOIs of liver, spleen, and kidney were manually delineated on PET images using the low-dose CT as a reference. For lung, VOIs were semiautomatically defined on the low-dose CT and projected on the PET images. Fixed-size VOIs of 7.5 and 1.6 mL were placed in the lumbar vertebrae to estimate bone marrow activity concentration and in the aortic arch to estimate blood-pool activity concentration.

### Plasma Concentration Analysis

Blood samples (plasma and serum) for ^89^Zr-GSK2849330 were obtained before dosing and at 1, 3, 6, 12, and 24 h after injection and immediately before PET scans. Plasma concentrations of ^89^Zr-GSK2849330 were assessed by radioactivity measurements in a cross-calibrated γ-counter (Wallac Wizard 1480; PerkinElmer Inc.) and expressed as SUV by normalization for infused activity and body weight. Unlabeled plasma GSK2849330 was quantified using a validated electrochemiluminescent immunoassay (21), with a concentration range of 50–5,000 ng/mL.

### Modeling of Radioactivity Uptake Data

**FIGURE 2.**
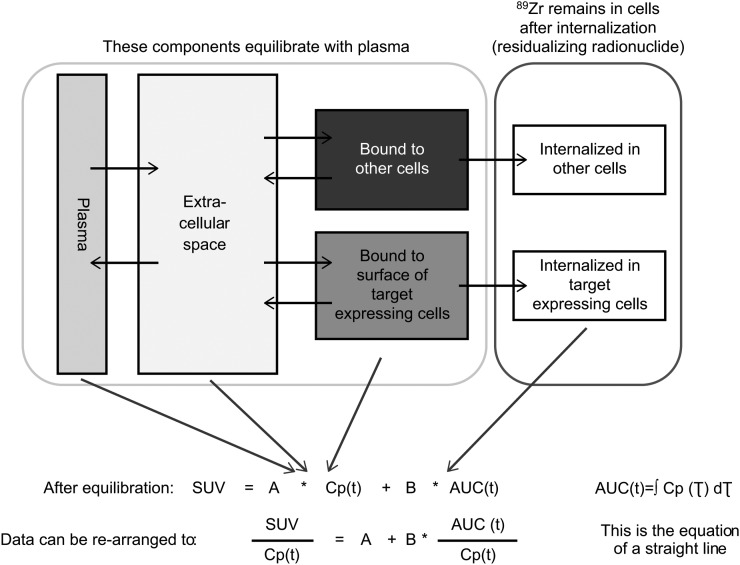
Schematic illustration of compartment model for components of tissue radioactivity and Patlak modeling. Cp(t) = radioactivity concentration; SUV = standardized uptake value.

A compartment model was used to represent tissue uptake of radioactivity (SUV; Fig. 2). In this model, 1 component of the tissue signal reflects radiolabeled mAb in plasma and interstitial space, which after a relevant time is in equilibrium with plasma plus radiolabeled mAb reversibly bound to the HER3 receptor or nontarget binding sites; after equilibration, this component is proportional to plasma radioactivity concentration. The other component of the tissue signal reflects irreversible internalization/residualization of ^89^Zr-mAb, which is dependent on receptor-binding properties. Assuming that internalization dominates over residence on the receptor, we postulate that internalization rate is proportional to the interstitial concentration; therefore, after equilibration, cumulative internalization is proportional to the AUC of the plasma radioactivity concentration. Further assumptions of the Patlak model have been previously described (22,23).

With these assumptions, the tissue signal SUV can be defined as a function of plasma radioactivity concentration *Cp*(*t*) using the following equation:

Eq. 1 $$\text{SUV}=A^{*}Cp\left(t\right)+B^{*}\overset{t}{\underset{0}{\int }}Cp\left(\tau \right)d\tau ,$$

From a kinetic standpoint, the constant *A* is related to radiolabeled mAb distribution in tissue, including plasma and a passive distribution in interstitial tissues plus reversible binding to target receptor and nontarget binding sites, whereas *B* is related to irreversible binding followed by internalization after binding to HER3 receptors plus nonspecific internalizing binding, such as pinocytosis.

Equation 1 can be rearranged as:

Eq. 2 SUV/Cp(t)=A+B×AUC(t)/Cp(t),

which describes a straight line with slope = *B* and intercept = *A*.

This rearrangement of imaging data has been used previously in PET imaging studies and is known as a Patlak plot (22,23). This study used Patlak plots to determine the slope (*B*) values by linear fit of individual PET data plotted at 2–3 time points.

### Modeling of Dose-Dependent Target Engagement

Each tumor is expected to have a different level of HER3 expression. Therefore, the derived parameters at different antibody mass doses were normalized and expressed as percentages of baseline 8-mg values. The *B*-value (slope) obtained after dosing of ^89^Zr-GSK2849330 in each patient was calculated as a percentage of the corresponding *B*-value from the baseline study. These relative values, which showed the internalization rates at increased mass doses compared with the baseline scan, were plotted against mass doses and were fitted to a theoretic dose-inhibition curve, with the 8-mg dose internalization rate set to 100%. The formula used for the fit comprised a constant term, which is thought to indicate nonspecific internalization, such as pinocytosis, and a dose-dependent term:

Eq. 3 $$R\left(\mathrm{dose}\right)=\left[1-\text{dose}/\left(\mathrm{dose}+ID_{\mathrm{50}}\right)\right]\times \left(\mathrm{100}-\mathrm{Rnsp}\right)+\mathrm{Rnsp,}$$

where *R* is the ratio of “postdose B-value” to “predose B-value,” expressed as a percentage, and Rnsp is the expected percent ratio at total inhibition of HER3 receptor internalization.

Equation 3 allows for evaluation of the 50% inhibitory mass dose (ID_50_) of target-mediated uptake of ^89^Zr-mAb by unlabeled mAb.

### Safety

Safety assessment included physical examinations, vital signs, 12-lead electrocardiograms, echocardiograms, clinical laboratory tests, and monitoring for protocol-defined adverse events (AEs) and serious AEs. The presence of anti-GSK2849330 antibodies was tested using a validated electrochemiluminescent immunoassay (21). Organ and whole-body radiation exposure were calculated using non–decay-corrected organ and blood radioactivity concentrations using the OLINDA program (24).

### Statistical Analysis

No formal hypotheses were tested for the primary endpoint. Point estimates and corresponding 95% confidence intervals by dose and site were constructed for absolute changes or percentage of changes from baseline in SUV_peak_, SUV_mean_, and volume of the region of interest.

## RESULTS

### Patients

**FIGURE 3.**
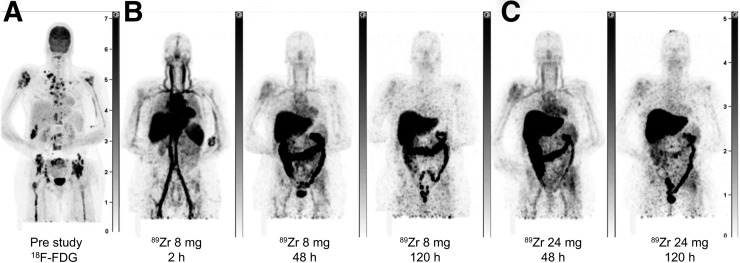
Maximum-intensity projections of patient 1: prestudy ^18^F-FDG scan (A), ^89^Zr-GSK2849330 PET after 8 mg total mAb dose at 2, 48, and 120 h after infusion (B), ^89^Zr-GSK2849330 PET after 24 mg total mAb dose at 48 and 120 h after infusion (C).

**FIGURE 4.**
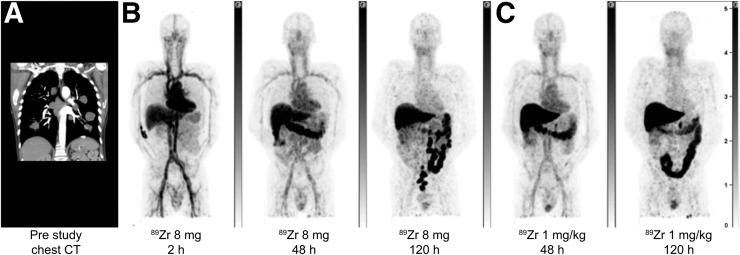
Maximum-intensity projections of patient 2: prestudy CT scan showing multiple lung lesions (A), ^89^Zr-GSK2849330 PET after 8 mg total mAb dose at 2, 48, and 120 h after infusion (B), ^89^Zr-GSK2849330 PET after 24 mg total mAb dose at 48 and 120 h after infusion (C).

Baseline characteristics for the 6 enrolled patients, as well as mAb doses for imaging, are summarized in Table 1. Drug plasma elimination and imaging in patients 1 and 2, who received total mAb doses of 8 mg and 24 mg at baseline (day 0) and 24 mg and 1 mg/kg on day 14, respectively, suggested that 8 mg of ^89^Zr-GSK2849330 was an appropriate imaging dose (Figs. 3 and 4 and Supplemental Table 1A; supplemental materials are available at http://jnm.snmjournals.org). At the 8-mg dose, circulating ^89^Zr-GSK2849330 remained in the blood over time, as evidenced by radioactivity in the heart and major blood vessels, with a constant and limited amount of tracer in the liver independent of total mAb mass dose (known as the sink effect; Supplemental Fig. 1 and data not shown). Note that plasma clearance was discretely reduced with the added mass doses of unlabeled mAb. Variable amounts of radioactivity were observed in the intestine, consistent with biliary excretion of tracer. All subsequent patients received a baseline mAb mass dose of 8 mg of ^89^Zr-GSK2849330 (Table 1). Subsequent total mass doses of 0.5 mg/kg (patient 5), 1 mg/kg (patient 2), and 30 mg/kg (patients 3 and 4) were administered on day 14. Patient 6 was discovered to have a brain metastasis in the baseline immuno-PET study and was removed from further studies. After completion of imaging procedures, patients continued treatment with GSK2849330 at the current safe dose in the first-time-in-human study.

**TABLE 1:** Patient Characteristics

Patient	Primary tumor type	mAb first/second administration	H-score of membrane staining	Best response*
1	Breast cancer	8 mg/24 mg	35	PD
2	Head and neck cancer	24 mg/1 mg/kg	80	ND
3	Cervical cancer	8 mg/30 mg/kg	184	PD
4	Ovarian cancer	8 mg/30 mg/kg	195	PD
5	Prostate cancer	8 mg/0.5 mg/kg	200	SD
6	Colorectal cancer	8 mg/—^†^	18	ND

* First response evaluation was performed at 12 wk after treatment initiation to allow sufficient time for beneficial treatment effect that could be detected by RECIST 1.1.

† Patient 6 was discovered to have brain metastasis as seen in baseline immuno-PET study and therefore was removed from further studies.

SD = stable disease; PD = progressive disease according to RECIST 1.1; ND = not determined.

### ^89^Zr-GSK2849330 PET Images

**FIGURE 5.**
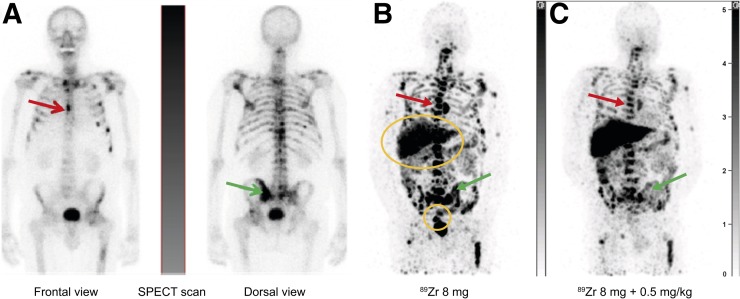
Maximum-intensity projections of patient 5 with osseous metastases of prostate cancer: SPECT scan after administration of ^99m^Tc-hydroxydiphosphonate (A), PET scans showing biodistribution and tumor accumulation of ^89^Zr-GSK2849330 at baseline with mass dose of 8 mg (day 5; after tracer injection only) (B) and on treatment with mass dose of 8 mg + 0.5 mg/kg unlabeled GSK2849330 (day 19, i.e., 5 d after tracer injection + unlabeled mAb) (C). Arrows point to examples of bone metastases; circles are over liver and rectum with ^89^Zr-GSK2849330-containing excretion.

PET imaging showed a dominant vascular distribution of radiolabeled mAb early after administration and a high uptake of ^89^Zr-mAb in the liver and spleen. Later PET scans (48 and 120 h after injection) indicated biliary excretion but also retention in major organs (e.g., liver) (Figs. 3– 5 and Supplemental Figs. 1 and 2). Day 5 after injection was chosen for quantification analysis of tumor uptake because this time point showed the best tumor-to-background contrast visually.

Tumor accumulation was visually observed in all patients to different extents, with SUV_peak_ ranging from 1.3 to 15.7 after the first tracer administration and from 1.6 to 11.2 at the second administration (Table 2).

**TABLE 2:** SUV_peak_ for All Lesions and Time Points After First and Second mAb Administrations

	First mAb administration			Second mAb administration	
Parameter	First time point	Second time point	Third time point	First time point	Second time point
Patient 1					
Hours after injection	2	48	119	49	117
SUV_peak_					
Bone lesion	BLQ	2.49	3.12	2.30	2.90
Bone lesion	4.19	4.55	2.60	4.80	3.51
Soft-tissue lesion	6.47	5.42	3.14	4.59	3.53
Soft-tissue lesion	2.26	4.53	3.60	4.87	3.10
Bone lesion	4.32	5.64	3.88	6.90	5.95
Bone lesion	3.36	3.23	2.89	3.36	2.96
Patient 2					
Hours after injection	2	46	115	44	118
SUV_peak_, lung lesion	2.22	1.87	1.57	1.71	1.57
Patient 3					
Hours after injection	2	50	116	49	139
SUV_peak_					
Lung lesion	BLQ	2.19	1.26	3.59	3.78
Bone lesion	BLQ	3.92	4.83	2.85	3.13
Bone lesion	BLQ	5.30	5.87	5.78	6.20
Bone lesion	BLQ	5.45	6.12	5.49	4.87
Patient 4					
Hours after injection	2	51	116	45	115
SUV_peak_, soft-tissue lesion	NA	9.94	12.01	5.72	5.92
Patient 5					
Hours after injection	2	42	138	40	113
SUV_peak_					
Lymph node lesion	NA	3.69	3.84	3.11	3.97
Bone lesion		15.66	15.26	6.58	11.23
Lymph node lesion		4.45	4.34	3.32	3.95
Lymph node lesion		3.96	4.06	2.72	2.92
Lymph node lesion		3.22	2.64	4.22	3.41
Lymph node lesion		4.60	5.38	4.50	5.71

BLQ = below limit of quantification; NA = not applicable.

### Effect of Increased Mass Dose

The plasma radioactivity concentration showed a relatively fast drug clearance, especially at the lowest mass dose, with a slower clearance at higher doses (Supplemental Table 1).

**FIGURE 6.**
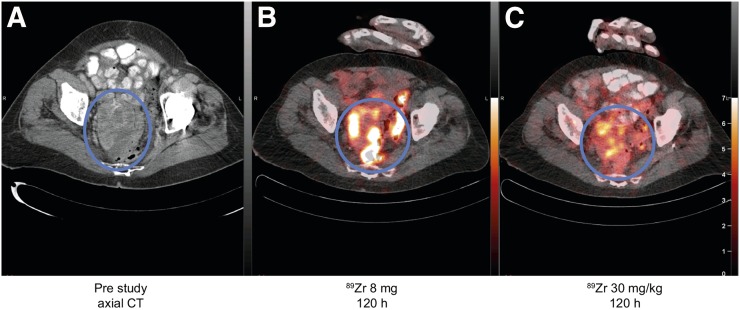
Images of patient 4: prestudy axial CT scan (A), ^89^Zr-GSK2849330 PET/CT after 8-mg total mAb dose at 120 h after injection (B), ^89^Zr-GSK2849330 PET/CT after 30 mg/kg total mAb dose at 120 h after injection (C). Circles highlight large metastasis in pelvic region. Both ^89^Zr-GSK2849330 PET images are normalized to same intensity.

**FIGURE 7.**
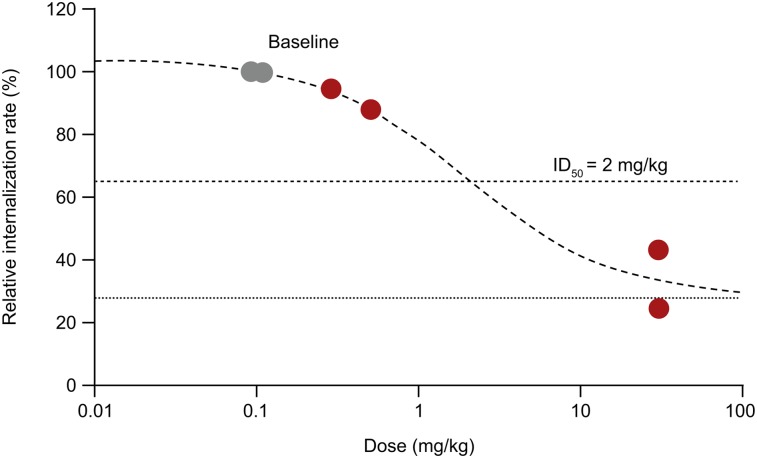
Modeling of tumor engagement with data from all patients. Gray circles indicate baseline studies normalized to 100%. Dotted line indicates expected nonspecific internalization, such as pinocytosis.

Reduced accumulation of radiolabeled mAb in tumor lesions after treatment, defined by SUV, was observed in patients 3 and 4 (Fig. 6 and Supplemental Figs. 2 and 3). To further quantify dose dependency, we performed a Patlak analysis to model irreversible tumor accumulation and observed a marked reduction in radiotracer accumulation rate at the highest dose of 30 mg/kg, suggesting saturation of the target, as seen in patients 3 and 4 (Supplemental Fig. 4). The fitted curve is compatible with an ID_50_ of 2 mg/kg and ID_90_ of 18 mg/kg of competing unlabeled mAb and a nonspecific tumor accumulation of 30% (Fig. 7). Because of limited data, no further statistical analyses were performed.

### Pharmacokinetic Results (Nonradioactive)

Across doses of 8–30 mg/kg, the maximum GSK2849330 (nonradioactive) plasma concentrations (C_max_) ranged from 2.1 to 1,515 μg/mL, and AUC from time 0 to 48 h (AUC_last_) ranged from 41.1 to 79,804 h × μg/mL. Exposure (C_max_ and AUC_last_) increased with increasing doses and, in general, was dose-proportional (data not shown). Time to C_max_ was between 1.1 and 2.4 h (data not shown). Plasma radioactivity pharmacokinetics, recalculated to mass concentration, and unlabeled pharmacokinetics had a constant ratio of 0.9 ± 0.19, suggesting consistency of disposition processes between labeled and unlabeled mAb (Supplemental Table 2 showing radioactivity and unlabeled pharmacokinetic data).

### Dosimetry

Radiation exposure was calculated for the first 4 patients, and the effective whole-body radiation dose was in the range of 17–22 mSv per 37 MBq, in line with previous studies (25) and predictions for the present study. Supplemental Table 3 shows a full listing of dosimetry data.

### Safety, Immunogenicity, and Response

The most-frequent AEs were decreased appetite (grade 1; 2 patients receiving 30 mg/kg in part 1) and diarrhea (grade 1; 2 patients receiving 30 mg/kg in part 2). One patient (patient 3) receiving 30 mg/kg experienced a protocol-defined serious AE of diarrhea during part 1, which was grade 3 in intensity, was considered drug-related, and resolved within 4 d. One patient (patient 2) receiving 30 mg/kg experienced a serious AE of arrhythmia during part 2 of the study, which was grade 3 in intensity, was considered drug-related, and resolved within 2 d. The patient was withdrawn from the study. Of the 5 patients who received GSK2849330 and provided an immunogenicity blood sample, 3 (60%) developed detectable levels of treatment-emergent anti-GSK2849330 antibodies.

The best response observed was stable disease at 12 wk in 1 patient (Table 1). There was no correlation between uptake measured by PET imaging (SUV) and response to treatment with GSK2849330.

## DISCUSSION

This study used immuno-PET imaging to track biodistribution of ^89^Zr-GSK2849330, an anti-HER3 antibody under development for treatment of HER3-positive tumors. ^89^Zr-mAb distribution was as expected, with predominantly vascular distribution early after tracer administration, accumulation in the liver, and significant tumor uptake later. We observed a trend toward higher ^89^Zr-GSK2849330 accumulation with higher HER3 expression. The excellent visualization of tumor lesions is likely, in part, a consequence of the PET technique, with its superior resolution and sensitivity compared with other nuclear modalities. Furthermore, the distinct HER3 tumor expression plus internalization and residualization of the radionuclide contributes to high contrast between lesions and most normal organs. Importantly, this study describes the dose-dependent inhibition of tumor uptake of ^89^Zr-mAb by unlabeled mAb confirming target engagement, that is, specific binding and internalization of anti-HER3 antibody by HER3 receptors in tumor tissues. It is important to consider what is meant by inhibition of accumulation rate and saturation. The analyses reveal the degree to which a small additional amount of mAb in plasma could bind to the HER3 receptor and be internalized if there was no target-mediated drug disposition (TMDD). Hence, it is a factor related to receptor functionality and not the amount of mAb that under TMDD conditions can access the tumors. This could be a relevant approximation of how the receptor system is saturated with respect to handling a constant level of heregulin. The SUVs by themselves represent capture of radioactivity by both specific and nonspecific phenomena, whereas Patlak analysis attempts to separate out the specific binding and internalization processes. These findings corroborate preclinical data reporting a dose-dependent, saturable uptake of ^89^Zr-GSK2849330 in HER3-positive (CHL-1) human xenograft tumors (12).

Compared with immunohistochemistry, showing HER3 expression in a biopsy at a single time point, immuno-PET imaging visualizes HER3 mAb binding and internalization of the whole body and over time. Thus, immuno-PET allows in vivo investigation of mAb pharmacokinetics, such as dose-dependent inhibition of tumor uptake. Indeed, with the highest mass dose of 30 mg/kg, we observed reduced uptake of ^89^Zr-GSK2849330 as measured by SUV_peak_ in tumor lesions (patients 4 and 5). In patient 1, no changes were observed, as expected with a similar mass dose (8 vs. 24 mg). Using the Patlak approach, we see saturation of irreversible HER3 accumulation with increasing mass dose in patients 3 and 4. Patient 2 did not show saturation of reversible or irreversible HER3 binding, possibly because of the relatively low HER3 expression (the relatively low SUV_peak_ is consistent with this hypothesis). Additionally, interpatient and intralesion heterogeneity in uptake of radiolabeled antibodies, as described previously (26), can lead to the observed heterogeneity in tracer uptake. Overall, our results are compatible with the saturation of HER3 by increasing unlabeled mAb supporting target engagement of GSK2849330 to HER3 receptors in the tumor tissues, with an estimated ID_50_ of 2 mg/kg. This fits well with the preclinically determined ID_50_ of 0.5 mg/kg (data not shown). Target saturation is expected to be achieved at ID_90_ and is estimated to be approximately 18 mg/kg based on the current Patlak model, which is considerably lower than the dose identified with the maximum-tolerated-dose approach (i.e., 30 mg/kg). However, because of the limited number of patients, these values are only an estimation and no statistical analysis can be performed.

A recent study with ^89^Zr-lumretuzumab, another anti-HER3 mAb, failed to show saturation of HER3 binding with increasing mass doses of mAb, despite using maximum doses similar to those in this study (27). Possibly, the binding epitope of the 2 mAbs differs, resulting in different levels of internalization and degradation of the HER3 receptor. However, they showed a near-complete downregulation of HER3 in serial skin biopsies, suggesting that saturating doses of HER3 binding could be achieved with lumretuzumab. Alternatively, our approach of serially imaging the same patient may be more informative in cases of variable tumor tracer accumulation between patients (as in this study), as baseline binding characteristics can be compared with those on treatment. Potentially, HER3 downregulation due to first tracer administration could interfere with imaging on treatment. However, although no on-treatment (skin) biopsies were available for immunohistochemistry of the HER3 receptor, baseline mAb mass dose was extremely low, making such an effect unlikely.

^89^Zr-GSK2849330 biodistribution showed significant uptake of ^89^Zr-mAb in the liver and spleen. This finding is likely due to both increased blood flow and Fc fragment of immunoglobulin G receptor IIIa binding (28). In addition, high tracer accumulation in normal liver tissue may also be due to catabolism, as 16% of immunoglobulin G is eliminated via the liver and subsequently excreted (29). This is in the same range as the average percentage injected dose found in the liver, that is, 20% (data not shown). Similar findings were observed in the lumretuzumab study, which reported the highest uptake of ^89^Zr-lumretuzumab in the liver and intestine (27). A methodologic aspect that strengthens confidence in this study is the good agreement between the plasma pharmacokinetics of the antibody and the equivalent concentration calculated through the SUVs in plasma (Supplemental Table 2).

Clinical trials in other tumor types have shown the potential of ^89^Zr-immuno-PET as a means of directly visualizing mAb target engagement (14,27,30). This present study is, to our knowledge, the first immuno-PET study describing target saturation in vivo in humans using an intricate design involving multiple tracer administrations and imaging time points. Despite the small patient cohort, this study demonstrates the feasibility of such an approach and provides dosing guidance for further evaluation of mAbs.

Patlak modeling was applied to determine the amount of drug internalized, taking into consideration the dose dependence of the plasma radioactivity pharmacokinetics (TMDD). This approach has the advantage of both normalizing the observed TMDD and providing relevant information with a limited number of scans. However, verification of the model assumptions was made difficult by logistical issues, such as appropriate selection of time points to ensure analysis of the linear part of the Patlak graph. Significant variability is eliminated by serial imaging of the same patients with different mAb doses, allowing for enrollment of fewer patients. We acknowledge that, because of its limited sample size, this study provides a demonstration of the conceptual design and feasibility of our approach rather than a robust statistical outcome. Patients imaged with intermediate mass doses between 0.5 and 30 mg/kg would have established more precise estimates of ID_50_ and ID_90_.

## CONCLUSION

This immuno-PET study explored the biodistribution and dose-dependent tumor uptake of ^89^Zr-GSK2849330 in patients with advanced solid tumors expressing HER3. Modeling of the uptake kinetics of ^89^Zr-GSK2849330 in tumors revealed a dose-dependent inhibition of rate of accumulation, indicating saturation of HER3 receptor at the highest mAb doses. This demonstrates the potential for immuno-PET to directly visualize tissue drug disposition in patients and to noninvasively measure target engagement at the site of action, offering the potential for dose selection.

## DISCLOSURE

Mats Bergstrom reports personal fees from OMID Molecular Imaging Consultancy. Iain McSherry, Matthew Cleveland, Wasfi Al-Azzam, Liangfu Chen, and Chris Matheny are employees and stock holders of GSK. Deborah Smith also holds stocks in GSK. Immanuel Freedman was an employee of GSK at the time of the study and held stocks in GSK. He is currently the sole proprietor of Freedman Patent. He has received consulting fees from Projections Research Inc. Sean Zhang was a previous employee of GSK. This work was supported by GlaxoSmithKline (ClinicalTrials.gov identifier NCT02345174). Editorial support, provided by Lisa Auker, PhD, and Ileana Stoica, PhD, of Fishawack Indicia Ltd., U.K., was funded by GSK. No other potential conflict of interest relevant to this article was reported.
