# Supplementary material for: Immuno-PET Imaging to Assess Target Engagement: Experience from 89Zr-Anti-HER3 mAb (GSK2849330) in Patients with Solid Tumors
Source: J Nucl Med. 2019 Jul;60(7):902–9. doi: 10.2967/jnumed.118.214726 (PMC6604691; doi:10.2967/jnumed.118.214726)
Supplement: Supplementary file 1 [file jnm214726SupplementalData.pdf]

Supplemental Figure 1. Biodistribution of 89Zr-GSK2849330 (A) before treatment imaged with tracer only (mass dose 8 mg) and (B) on treatment imaged with tracer and cold mAb (mass dose 8 mg + 30 mg/kg).

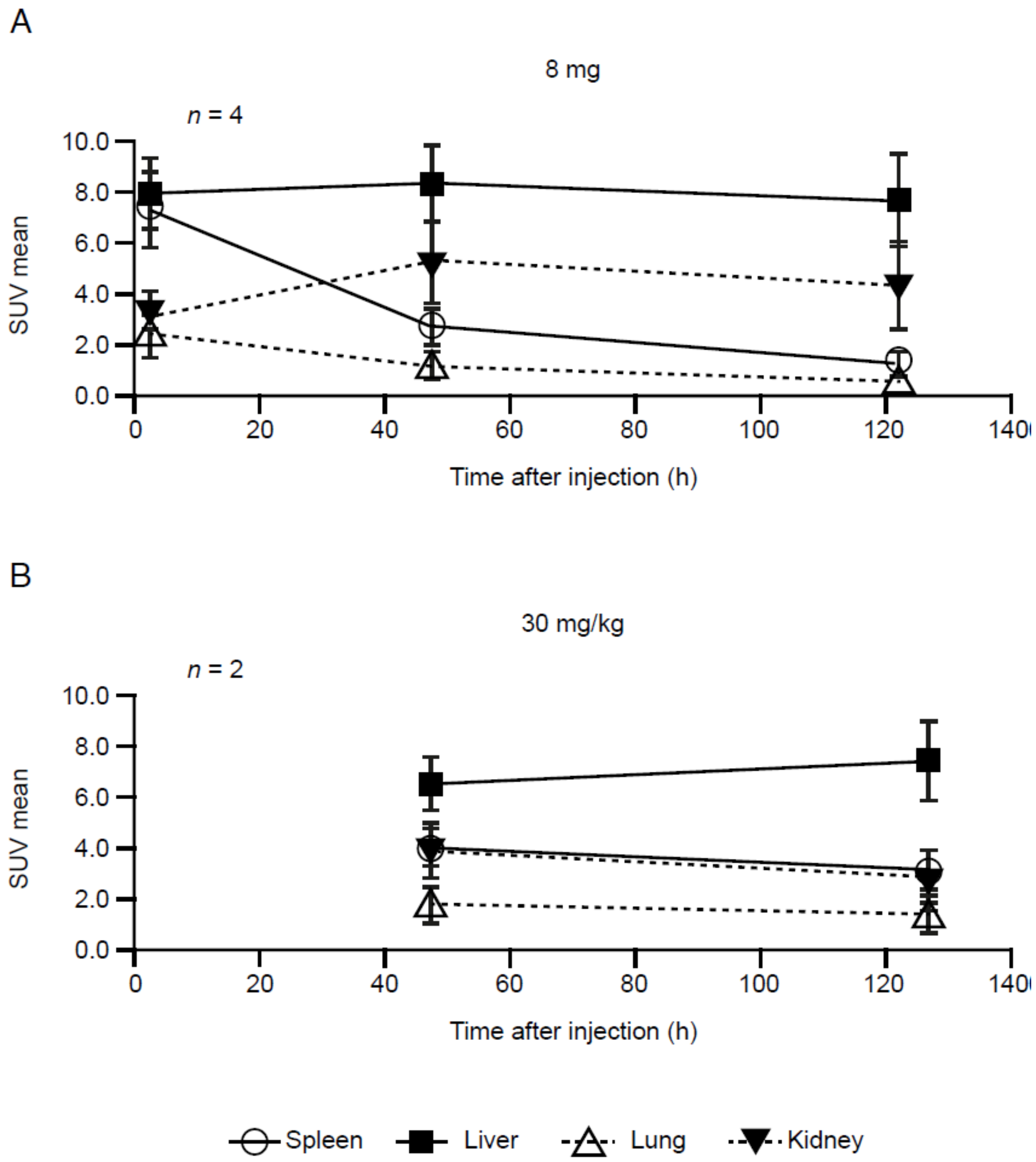

Error bars represent standard error of the mean.

mAb, monoclonal antibody; SUV, standardized uptake value.

Supplemental Figure 2. MIP images of patient 4 seen on (A) pre-study CT scan showing abdominal mass (red arrow); 89Zr-GSK2849330 PET following 8 mg total mAb dose at (B) 2 h, 48 h and 120 h p.i.; 89Zr-GSK2849330 PET following 30 mg/kg total mAb dose at (C) 48 h and 120 h p.i.

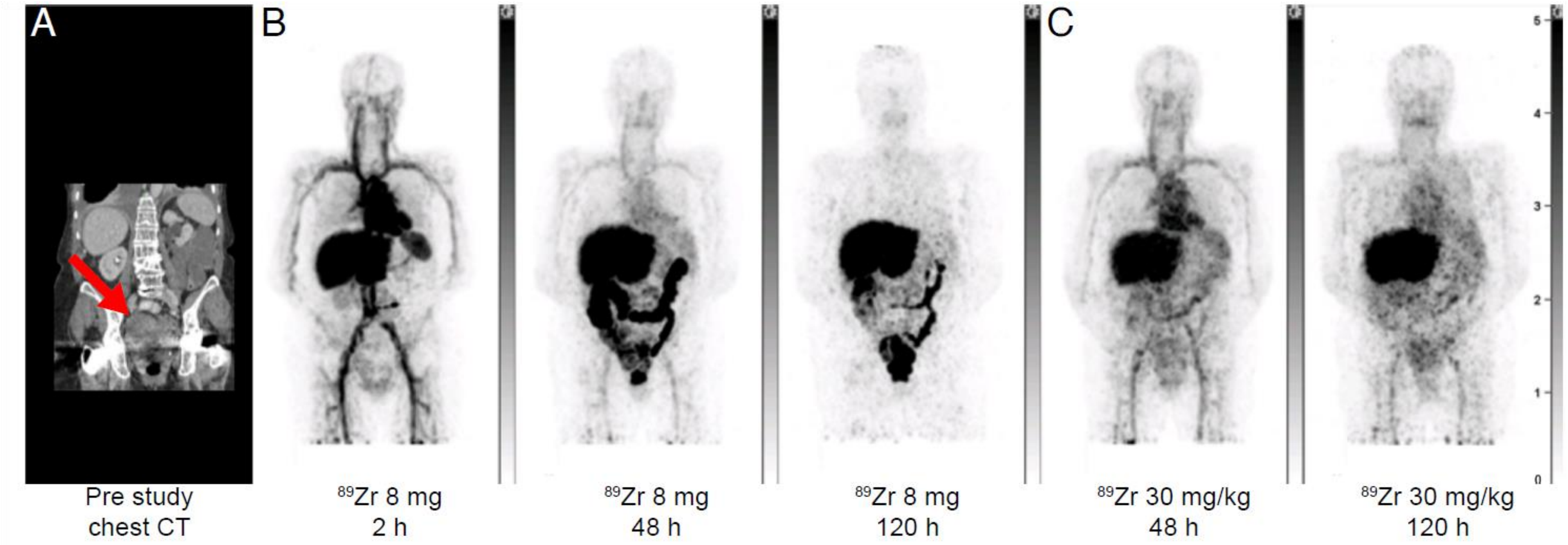

CT, computed tomography; mAb, monoclonal antibody; MIP, maximum intensity projection; PET, positron emission tomography; p.i., post infusion.

Supplemental Figure 3. (A) Average SUV_{peak} in tumor lesions and (B) SUV_{mean} plasma radio-pharmacokinetic values for all patients. Red lines indicate baseline (8 mg mass dose) and blue lines indicate follow-up with added mass doses of unlabeled mAb (8 mg + 30 mg/kg mass dose).

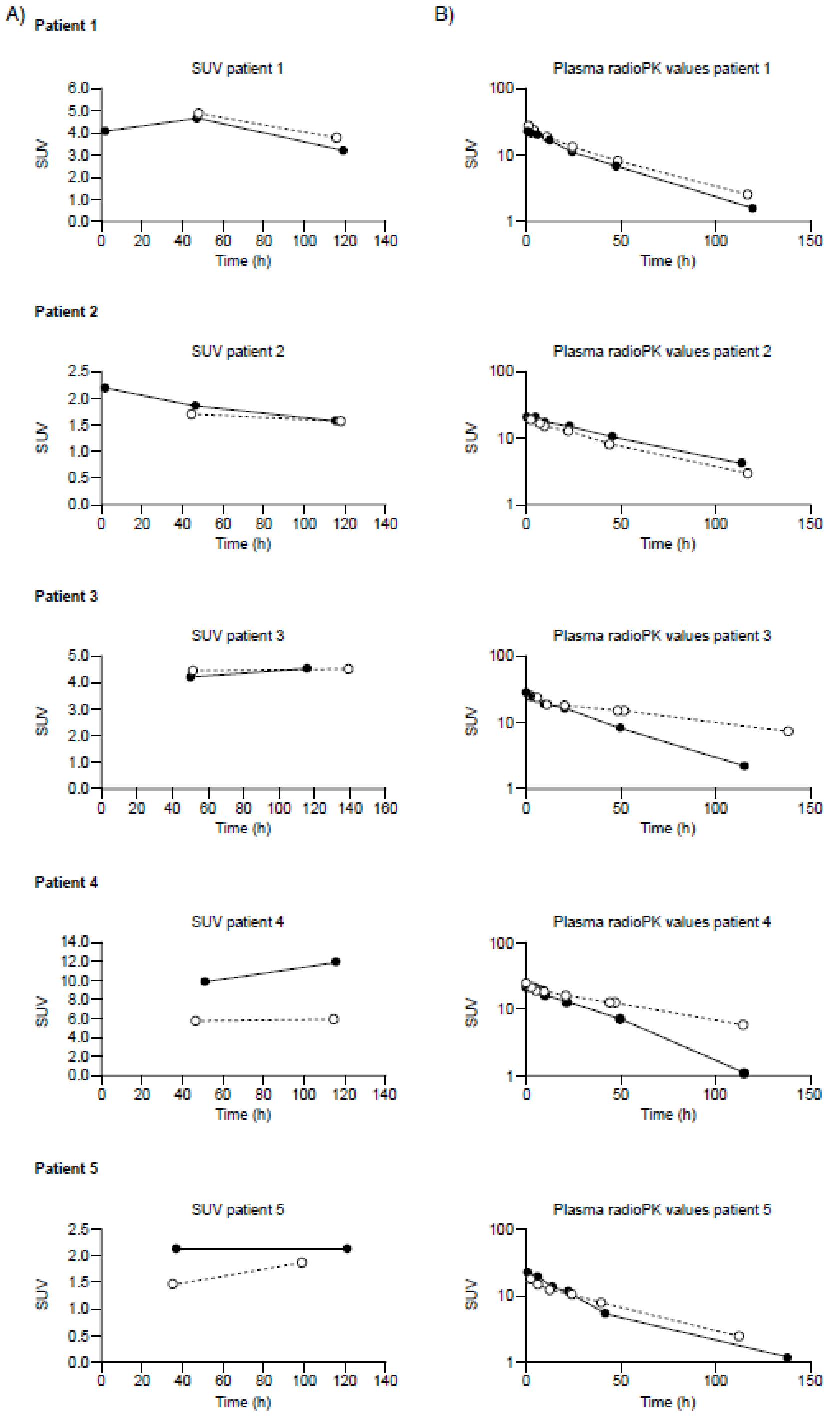

MAb, monoclonal antibody; PK, pharmacokinetic; SUV, standardized uptake value.

Supplemental Figure 4. Patlak plot for all patients. Patient 3 was given 30 mg/kg in the follow-up study. Red lines indicate baseline (8 mg mass dose) and blue lines the follow-up with added mass doses (8 mg +30 mg/kg mass dose).

A straight line was fitted to the graphs and the slope and intercept values were determined.

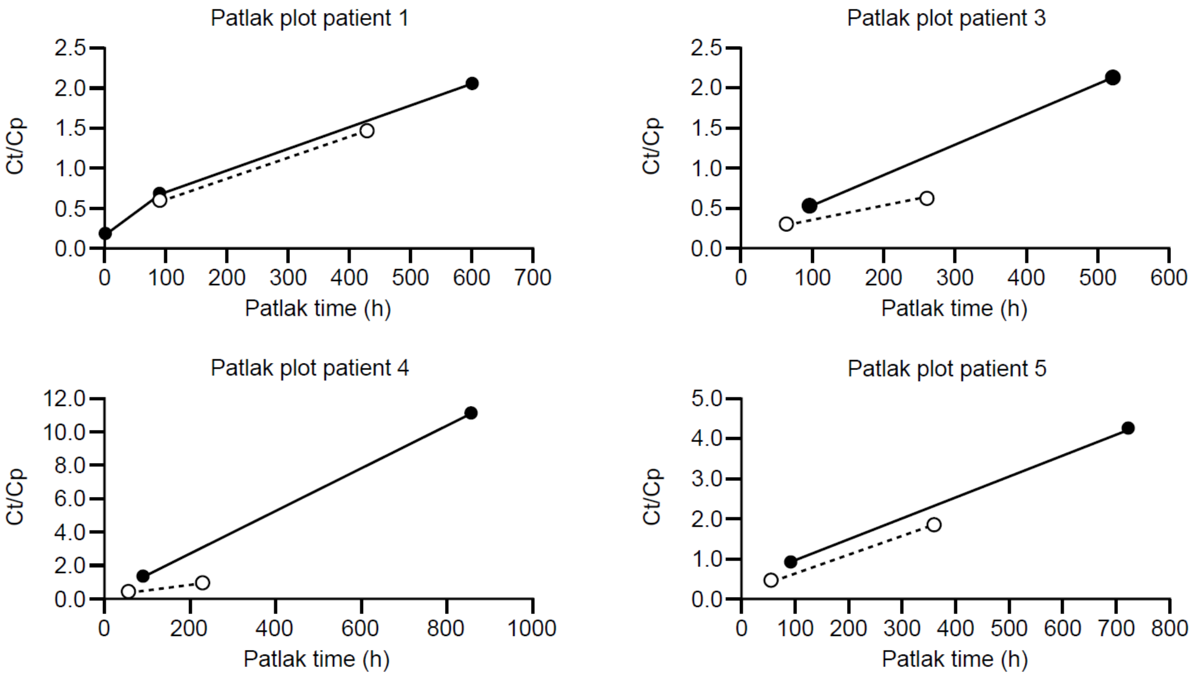

Supplemental Table 1. SUV values in plasma for all patients (Patients 1 and 2: Table 1a; Patients 3 and 4: Table 1b; and Patient 5: Table 1c). For reference, tumor SUV_{peak} values (see Table 2) are included.

a)

**Patient 1 first session 8 mg**			**Patient 1 second session 24 mg**			**Patient 2 first session 24 mg**			**Patient 2 second session 1 mg/kg**		
**Time (h)**	**Tumor**	**Plasma**	**Time (h)**	**Tumor**	**Plasma**	**Time (h)**	**Tumor**	**Plasma**	**Time (h)**	**Tumor**	**Plasma**
0.9		23.5	1.2		28.3	0.8		20.0	1.5		19.6
1.6		23.9	2.9		26.4	1.6		20.4	3.0		18.2
2.1	4.1	22.5	10.0		19.4	2.2	2.2	20.4	6.2		17.0
2.3		21.8	23.7		13.8	3.7		20.3	10.0		15.1
6.0		20.9	47.9		8.3	5.8		19.7	22.0		12.9
12.0		17.2	48.1	4.9	8.3	9.8		17.2	43.9		8.1
24.3		11.0	116.0		2.6	22.8		14.9	44.5	1.7	8.1
46.9		7.0	116.2	3.8	2.6	45.9		10.5	117.0		3.0
47.5	4.7	7.0				46.3	1.9	10.5	117.6	1.6	3.0
118.5		1.6				114.0		4.2			
119.2	3.2	1.6				114.6	1.6	4.1			

b)

Patient 3 first session 8 mg			Patient 3 second session 30 mg/kg			Patient 4 first session 8 mg			Patient 4 second session 30 mg/kg		
Time (h)	Tumor	Plasma	Time (h)	Tumor	Plasma	Time (h)	Tumor	Plasma	Time (h)	Tumor	Plasma
1.2		28.2	1.1		26.8	1.1		21.7	0.4		24.8
1.9		25.6							2.3		22.3
3.5		23.9	3.0		23.3	3.0		20.9			
5.2		24.3	6.7		24.1	6.0		19.4	5.4		19.4
10.2		18.7	12.0		18.3	10.0		16.9	9.2		18.5
21.5		16.0	21.1		17.7	21.5		12.9	20.9		16.2
49.9		8.1	48.7		14.9	50.1		7.1	44.4		12.7
50.4	4.2	8.1	51.8	4.4	14.6	50.7	9.9	7.1	46.5	5.7	12.5
114.8		2.2	137.9		7.3	114.9		1.2	114.0		6.0
115.6	4.5	2.1	139.0	4.5	7.2	115.7	12.0	1.1	114.5	5.9	5.9

c)

Patient 5 first session 8 mg			Patient 5 second session 0.5 mg/kg		
Time (h)	Tumor	Plasma	Time (h)	Tumor	Plasma
1.1		22.3	1.0		18.8
3.0		18.1	3.0		17.9
6.2		19.6	6.0		15.1

13.7		13.8	12.0		12.5
22.4		11.6	23.8		10.8
41.6		5.6	39.3		8.1
42.2	5.9	5.6	39.9	4.1	8.0
137.5		1.2	111.3		2.5
138.1	5.9	1.2	113.1	5.2	2.4

h, hours; SUV, standardized uptake value.

Note: Patient 6 had only “baseline” scans and therefore did not have data available to be shown here.

Supplemental Table 2. Plasma radioactivity **(a)** and unlabeled **(b)** pharmacokinetic parameters.

a)

**Patient**	**1**		**2**		**3**		**4**		**5**	
**Dose (mg)**	8	24	24	1 mg/kg	8	30 mg/kg	8	30 mg/kg	8	0.5 mg/kg
**Initial DV (%)**	5.5	5.0	5.0	5.9	4.2	4.8	4.2	4.9	6.7	6.4
**Half-life (h)**	34.7	38.5	49.5	46.2	33.0	93.6	26.7	63.0	26.7	43.3

Only data after 10 hours were used in the calculations.

b)

		**8 mg hot infusion**	**24 mg hot infusion**	**1 mg/kg (hot + cold infusion)**	**30 mg/kg (hot + cold infusion)**	**0.5 mg/kg (hot + cold infusion)**
**C_{max} (µg/L)**	Geometric mean (CVb%)	2671.3 (14.5)	11392.1 (8.8)	21060.9 (NA)	1193775.9 (34.7)	9558.8 (NA)
	Min, max	2137, 3192	10707, 12121	-	940715, 1514912	-
**T_{max} (h)**	Median	1.30	1.58	2.42	1.91	2.17
	Min, max	1.1, 1.8	1.4, 1.8	-	1.6, 2.3	-
**AUC_{INT} (h*µg/L)**	Geometric mean (CVb%)	43380.0 (22.3)	184594.3 (4.1)	331368.9 (NA)	20321852.0 (16.1)	161867.7 (NA)
	Min, max	30169, 54869	179326, 190017	-	18145259, 22759536	-
**AUC_{LST} (h*µg/L)**	Geometric mean (CVb%)	67719.5 (29.9)	438618.5 (19.8)	836335.9 (NA)	63951525.6 (32.1)	404624.1 (NA)
	Min, max	41122, 85337	381942, 503705	-	51247798, 79804358	-
**LAMZHL (h)**	Geometric mean (CVb%)	32.07 (62.1)	37.13 (21.2)	41.75 (NA)	61.79 (25.5)	34.63 (NA)
	Min, max	19.0, 81.4	32.0, 43.1	-	51.9, 73.5	-

AUC_{INT}, area under the concentration-time curve from time 0 to 24 hours; AUC_{LST}, area under the concentration-time curve from time 0 to last sample that is quantifiable; C_{max}, maximum plasma concentration; CVb, coefficient of variance; DV, distribution volume; LAMZHL, initial half-life; max, maximum; min, minimum; NA, not applicable; T_{max}, time to maximum concentration.

Hot infusion=89Zr-labeled GSK2849330; Cold infusion=unlabeled GSK2849330.

Supplemental Table 3. Listing of all dosimetry data (all treated population).

**Patient**	**1**	**2**	**3**	**4**
**Treatment**	**A/B/G/H**	**B/C/G/H**	**A/F/G/H**	**A/F/G/H**
First dose GSK2849330	8 mg	24 mg	8 mg	8 mg
Spleen (mSv)	0.2393	0.1005	0.1713	0.1789
Bone marrow (mSv)	2.2148	1.8538	2.2639	2.1775
Liver (mSv)	4.7867	2.5572	4.3840	4.4720
Lungs (mSv)	3.6470	3.2259	3.4250	3.2164
Kidney (mSv)	0.2165	0.1663	0.2535	0.1892
Body (mSv)	22.072	16.771	21.817	21.122

MBq, megaBequerel; mSv, milliSievert.

Dosimetry result in mSv units = Original result in mSv/MBq units *proper infused dose (in MBq) obtained as the amount in the syringe before administration minus the residual amount in the syringe after infusion.

A=8 mg Hot infusion; B=24 mg Hot infusion; C=1 mg/kg (Hot + Cold infusion); D=3 mg/kg (Hot + Cold infusion); F=30 mg/kg (Hot + Cold infusion); I=0.5 mg/kg (Hot + Cold infusion); G=30 mg/kg Cold infusion (once a week for 5 weeks); H=30 mg/kg Cold infusion (once every 2 weeks).

Hot infusion=89Zr-labeled GSK2849330; cold infusion=unlabeled GSK2849330.
